# Molecular detection of *Coxiella burnetii* in heart valve tissue from patients with culture-negative infective endocarditis

**DOI:** 10.1097/MD.0000000000011881

**Published:** 2018-08-24

**Authors:** Young-Rock Jang, Joon Seon Song, Choong Eun Jin, Byung-Han Ryu, Se Yoon Park, Sang-Oh Lee, Sang-Ho Choi, Yang Soo Kim, Jun Hee Woo, Jae-Kwan Song, Yong Shin, Sung-Han Kim

**Affiliations:** a Department of Infectious Diseases, Asan Medical Center, University of Ulsan College of Medicine, Seoul; b Division of Infectious Disease, Department of Internal Medicine, Gil Medical Center, Gachon University College of Medicine, Incheon; c Department of Pathology; d Department of Convergence Medicine, Asan Medical Center, University of Ulsan College of Medicine; e Division of Infectious Diseases, Department of Internal Medicine, Soonchunhyang University Seoul Hospital, Soonchunhyang University College of Medicine; f Department of Cardiology, Asan Medical Center, University of Ulsan College of Medicine, Seoul, Republic of Korea.

**Keywords:** diagnosis, endocarditis, polymerase chain reaction, Q fever

## Abstract

*Coxiella burnetii* is a common cause of blood culture–negative infective endocarditis (IE). Molecular detection of *C burnetii* DNA in clinical specimens is a promising method of diagnosing Q fever endocarditis. Here, we examined the diagnostic utility of Q fever polymerase chain reaction (PCR) of formalin-fixed heart valve tissue from patients with blood culture–negative IE who underwent heart valve surgery. Clinical and laboratory data of patients with blood culture–negative IE who underwent heart valve surgery during a 6-year period and for whom biopsy tissues were available were reviewed retrospectively. Blood culture–positive IE patients who underwent heart valve surgery within the last 3 years were used as controls. Heart valve samples were cultured and also subjected to histological examination and PCR for Q fever, brucellosis, and bartonellosis. Data from 20 patients with blood culture–negative IE and 20 with blood culture–positive IE were analyzed. Eight cases of blood culture–negative IE were PCR-positive for *C burnetii* (40%; 95% confidence interval, 19–64). No specimen was PCR-positive for brucellosis or bartonellosis. Histologically, 4 of 8 specimens with a positive Q fever PCR result were characterized by clusters of multinucleated giant cells without a fibrin ring. None of 20 patients with blood culture–negative IE received anti-*Coxiella* antibiotic therapy due to lack of clinical suspicion. Six-month mortality was higher in the Q fever PCR-positive group than in the Q fever PCR-negative group [38% (3/8) vs 0% (0/12), *P* = .049). Of the 20 patients with blood culture–positive IE, none yielded a positive Q fever PCR result for valve tissue. Approximately 40% of patients with culture-negative IE who received heart valve surgery were PCR-positive for Q fever; patients without clinical suspicion suffered high mortality. These data suggest that Q fever IE in patients with culture-negative IE is often missed in routine clinical practice.

## Abstract 2

Supplemental Digital Content is available in the text

## 1 Introduction

The incidence of blood culture–negative infective endocarditis (IE) ranges from 2.5% to 31%.^[1,2]^ There are several reasons for negative blood culture results in patients with suspected IE: subacute right-sided IE; cultures taken toward the end of a chronic course; uremia supervening in a chronic course; mural IE, as in ventricular septal defects, thrombi postmyocardial infarction, or infection related to pacemaker wires; slow growth of fastidious organisms; prior administration of antibiotics; fungal IE; IE caused by obligate intracellular parasites; or non-IE or an incorrect diagnosis.^[2–4]^*Coxiella burnetii* is a fastidious bacterium that causes blood culture–negative IE.^[5,6]^ Q fever endocarditis accounts for at least 5% of all IE cases and for 45% of culture-negative cases.^[6,7]^ Special diagnostic tests are not used routinely in all cases of IE, but may be useful for culture-negative IE; furthermore, molecular techniques to recover specific DNA from valve tissue samples have been useful diagnostically in selected cases.^[8]^ Recently, polymerase chain reaction (PCR) techniques have been developed for Q fever testing and were used successfully to detect DNA in clinical samples,^[9]^ and formalin-fixed tissues.^[10]^

Therefore, the aim of this study was to evaluate the utility of Q fever PCR of formalin-fixed cardiac valve tissue from patients with culture-negative IE who received heart valve surgery, and to investigate the clinical characteristics of Q fever IE diagnosed using this diagnostic test.

## 2 Methods

### 2.1 Study patients

The medical records of all patients admitted to Asan Medical Center with a diagnosis of IE from January 2001 to June 2016, and who had negative blood cultures and underwent valve replacement (and for whom biopsy tissue was available), were examined retrospectively. As far as possible, patients with culture-negative IE met the modified Duke's criteria^[11]^ based on gross features and histopathological findings. A diagnosis of IE was rejected if there was no pathological evidence to support it; such cases were excluded from the study. Patients with culture-positive IE and who underwent heart valve surgery within the last 3 years were also selected as controls. Excised cardiac valve tissues were cultured, formalin-fixed, and paraffin-embedded. Paraffin-embedded cardiac valves were stained with hematoxylin-eosin.^[12]^ In addition, tissues samples were stained with Giemsa, Gram periodic-acid Schiff, Grocott-Gomori, Warthin-Starry, Gimenez, and Ziehl-Neelsen to detect microorganisms.^[13]^ A culture-negative result was defined as no growth of microorganisms in blood cultures or from cardiac valve tissues, and absence of microorganisms upon histologic examination. This study was approved by the Asan Medical Center Institutional Review Board.

### 2.2 Molecular methods

#### 2.2.1 DNA extraction

To detect *C burnetii*, DNA was extracted from formalin-fixed cardiac valve tissues. Five sections (5 μm thick) were cut from each paraffin block and placed in a microtube. First, xylene was added, the tube was centrifuged (12,000 rpm, 5 minutes), and the supernatant was discarded. This procedure was repeated 3 times. The specimens were then rehydrated through a graded series of ethanol solutions and centrifuged after each washing step. Finally, the tubes were kept open to allow any remaining ethanol to evaporate. DNA was extracted using a QIAamp DNA Mini Kit (Qiagen, Hilden, Germany), according to the manufacturer's instruction with minor modifications. Briefly, tissue was digested in AL buffer and proteinase K (samples were kept in a water bath for 18 hours). Samples were washed twice in AL buffer, and DNA was eluted in 100 μL of Tris-Acetate EDTA (TAE) buffer and stored at −20°C until use.

#### 2.2.2 PCR assay

End-point PCR was performed to detect *C burnetii* in tissue samples. The gene target was derived from the transposase gene insertion element *IS1111a* of *C burnetii* isolate LBCE 13265 (NCBI Nr. KT 965031.1). The forward (5’-GAGCGAACCATTGGTATCG-3’) and reverse (5’-TTTAACAGCGCTTGAACGT-3’) primers were synthesized at the usual length of around 24 bp. The end-point PCR process comprised an initial denaturation step at 95°C for 15 minutes, followed by 45 cycles at 95°C for 30 seconds, 57°C for 30 seconds, and 72°C for 30 seconds, and a final elongation step at 72°C for 7 minutes. DNA (5 μL) was amplified in a total volume of 25 μL containing 10× PCR buffer (Qiagen), 2.5 mM MgCl_2_, 0.25 mM deoxynucleotide triphosphate, 25 pmol of each primer, and 1 unit of Taq DNA polymerase (Qiagen). PCR products were separated in 2% agarose gels containing ethidium bromide and visualized using a GelDoc System (Clinx Science Instruments, Shanghai, China).

#### 2.2.3 Sequencing of PCR products

For direct sequencing of DNA, all samples were amplified with primers specific for *C burnetii* and then purified using Expin PCR SV (GeneAll, Seoul, Korea). Purified samples were sequenced directly using BigDye Terminator chemistry and forward primer Q-fever_IS111. Sequencing was performed by Macrogen sequencing service (Macrogen Inc, Seoul, Korea), which examined the DNA sequencing reactions on an ABI 3730XL DNA Analyzer (Applied Biosystems, Foster City, CA, USA), which produces read lengths of 800 to 1000 bases.

### 2.3 Statistical analysis

Data from patients with Q fever endocarditis were compared with those from patients in the non-Q fever IE group using 2-sample *t* tests (continuous variables) or Chi-square/Fisher exact tests (categorical variables). Differences were considered significant at *P* < .05. All calculations were performed using SPSS for Windows software package, version 21 K (SPSS Inc, Chicago, IL).

## 3 Results

**Figure 1:**
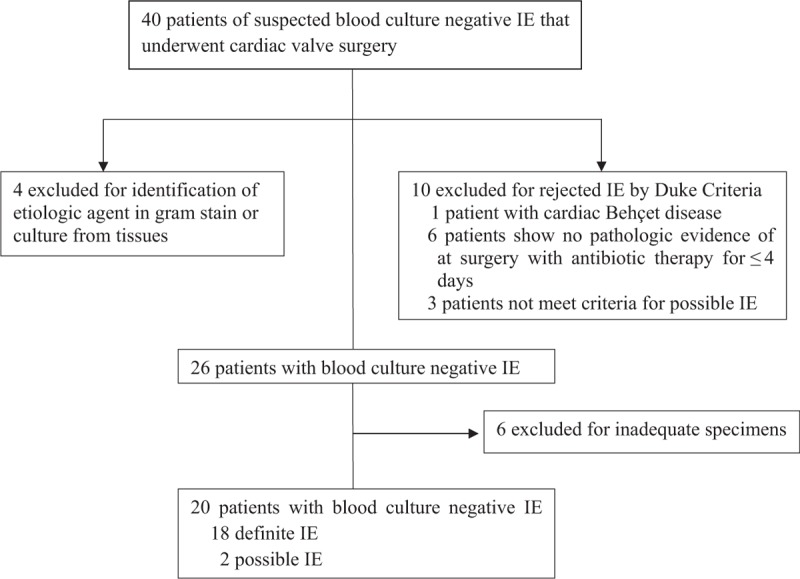
Distribution of the 40 patients with suspected blood culture–negative infective endocarditis who underwent cardiac valve surgery from January 1, 2011 to July 31, 2016 (according to the Duke criteria and etiological diagnoses). IE = infective endocarditis.

Specimens from 40 patients with suspected blood culture–negative IE who underwent cardiac valve surgery were included. Application of the modified Duke criteria^[11]^ led to a rejection of IE in 10 (25%) patients, including 1 (3%) with cardiac Behçet disease (Fig. 1).^[14]^ Of the remaining 30 patients with IE, 4 (13%) were excluded due to an infectious agent identified on tissue culture or histologic examination. Of the remaining 26 patients with a diagnosis of culture-negative IE, 6 were excluded due to lack of sufficient material for analysis. Finally, the remaining 20 patients were classified as culture-negative IE (Fig. 1). The baseline clinical characteristics, indications for surgery, and histopathologic findings are shown in Tables 1 and 2. The median age (range) was 55 years (43–71), and the majority of patients were men (men:women, 14:6). Five patients (25%) had a valvular prosthesis. All patients received empirical antibacterial therapy (ceftriaxone, cefepime, nafcillin, gentamicin, or vancomycin), and none received anti-*Coxiella* antibiotic therapy.

**Table 1:**
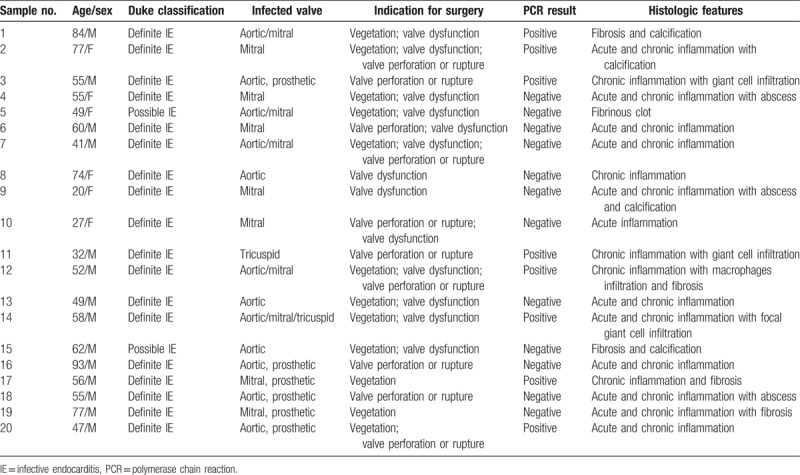
Results of Q fever polymerase chain reaction of heart valve tissue from 20 patients with culture-negative infective endocarditis.

**Table 2:**
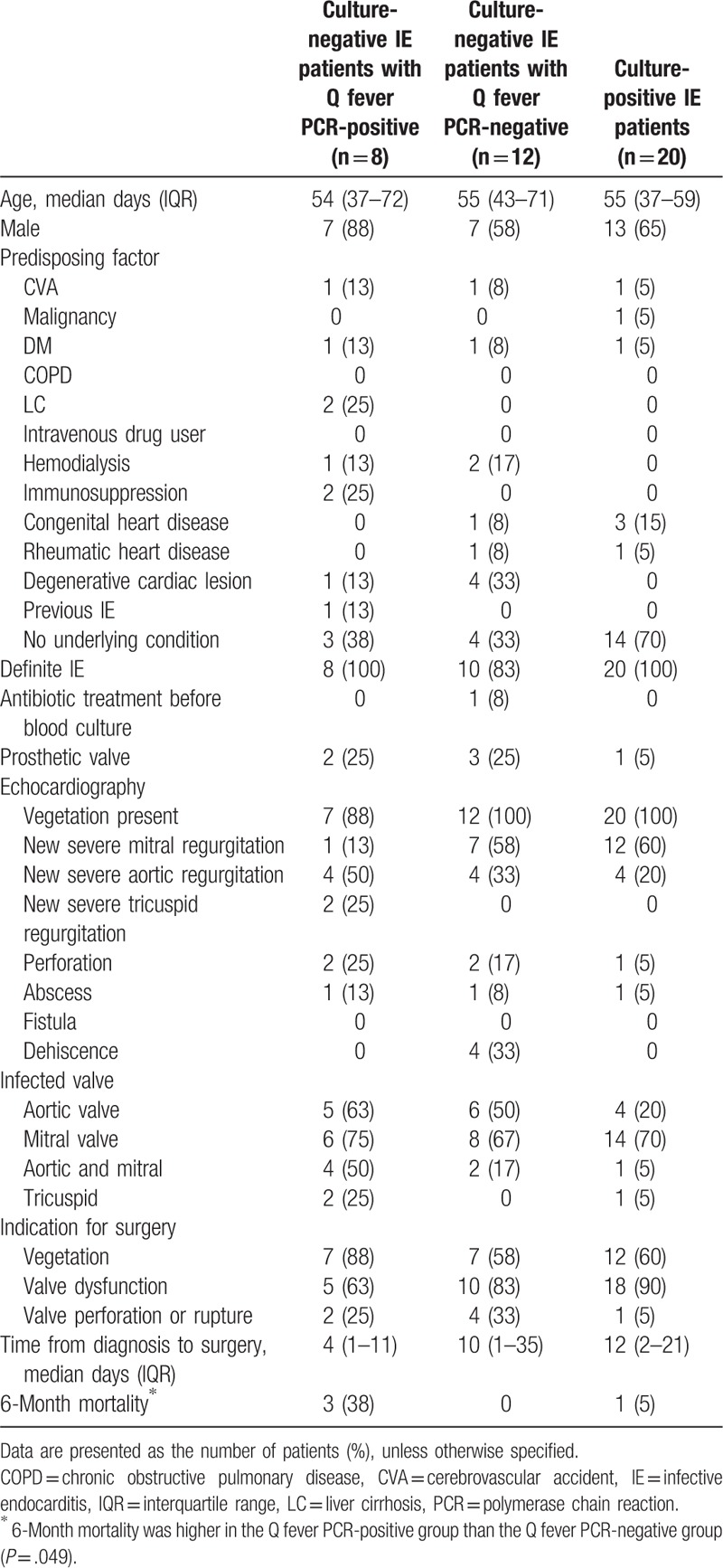
Clinical characteristics of patients with infective endocarditis treated surgically.

**Figure 2:**
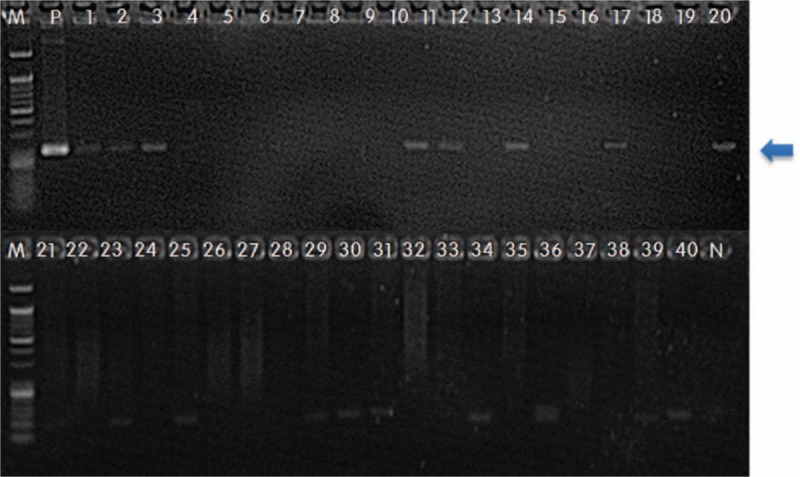
Agarose gel electrophoresis of polymerase chain reaction (PCR) products derived from the *Coxiella burnetii IS1111a* gene. Amplification of bacterial DNA using Q fever-*IS1111a* primers to detect *C burnetii*. Gel electrophoresis of end-point PCR products (202 bp). M: 50 bp DNA size marker; 1–20: DNA samples from patients with culture-negative infective endocarditis (IE); 21–40: DNA samples from patients with culture-positive IE and the negative controls (N).

PCR was performed to detect the *C burnetii* in all specimens from the 20 patients included the study. Of these, 8 (40%; 95% confidence interval, 19–64) had positive results. An agarose gel profile of the PCR products from these patients is presented in Figure 2. No specimen was PCR positive for brucellosis and bartonellosis. Histological examination of valve tissue from 4 (50%) of these 8 patients revealed clusters of multinucleated giant cells without a fibrin ring; this feature was not observed in samples from the Q fever PCR-negative group (Supplemental Fig. 1). Because of lack of clinical suspicion, only 2 patients (25%) underwent Q fever serology tests. No phase I or II antibodies were detected in 1 patient at 2 weeks postadmission. No phase II antibody was detected in the other patient at 1 week postadmission. Neither patient underwent serological follow-up. All-cause 6-month mortality was higher in the Q fever PCR-positive group than in the Q fever PCR-negative group (38% vs 0%; *P* = .049).

Twenty controls with blood culture–positive IE were evaluated to check for potential false-positive results. All the patients met the Duke criteria for IE. *Viridans* group streptococci was the most common cause of IE (n = 16), followed by *Staphylococcus aureus* (n = 2), and *Hemophilus* species, *Aggregatibacter* species, *Cardiobacterium hominis*, *Eikenella corrodens*, and *Kingella* species organisms (n = 2) (Table 3). Of the 20 control patients, none showed a positive Q fever PCR result from cardiac valve tissue. The agarose gel profile of the PCR products is shown in Figure 2.

**Table 3:**
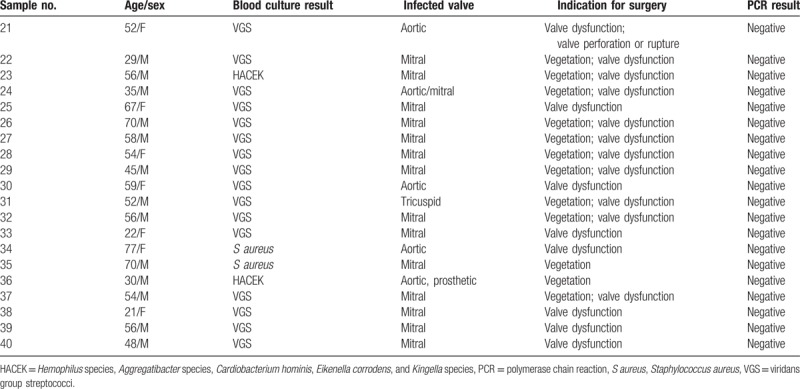
Results of Q fever polymerase chain reaction of heart valve tissue in 20 control patients with a definitive diagnosis of infective endocarditis.

## 4 Discussion

Failure to culture microorganisms that cause IE is a major problem that complicates diagnosis and prevents timely and effective treatment. Q fever IE is one of the most common causes of culture-negative IE (second only to prior antibiotic use). Q fever IE is, however, usually missed in real clinical practice because the clinical manifestations are nonspecific and serology tests for *C burnetii* have a wide range of positive-predictive values.^[15]^ In fact, vegetations are either small or absent from more than one third of Q fever IE cases^[15,16]^ and valvular inflammation is insignificant,^[16]^ further complicating clinical diagnosis. In this context, documentation of Q fever IE in pathologic specimens plays an important role in a definitive diagnosis. Indeed, Li et al^[11]^ proposed that positive culture, PCR, or immunohistochemical analysis of cardiac valve tissue is a definitive criterion for Q fever IE. Here, the PCR results showed that Q fever endocarditis accounted for approximately 40% of all culture-negative IE cases; this is consistent with previous reports.^[6,17]^ In addition, none of the patients with positive Q fever PCR results for cardiac valve tissue received anti-*Coxiella* therapy, and mortality was higher than that for culture-negative IE patients who had negative Q fever PCR results. In most instances, patients who presented as culture-negative IE were not suspected as having Q fever IE by clinicians, and a substantial proportion of patients with Q fever IE were misdiagnosed and suffered a poor outcome. Therefore, further studies are needed to examine the epidemiology and outcomes of “missed” Q fever IE patients.

There are some concerns about the value of PCR-based tests. A previous study suggests that blood Q fever PCR should be considered as a major criterion rather than a definitive one^[15]^; however, direct detection of targets at the disease sites using molecular methods is considered a definite diagnosis. Our previous study showed that PCR targeting of a *C burnetii* IS1111 multicopy sequence from formalin-fixed, paraffin-embedded liver tissues is useful for diagnosing patients with suspected Q fever.^[10]^ Therefore, we believe that PCR or immunohistochemical analysis of cardiac valves for Q fever should be included as a major criterion in the Duke endocarditis criteria (in addition to pre-existing serologic criteria).

Vegetation occurs in 21% to 50% of patients with Q fever endocarditis^[12,15]^; however, we found vegetations in excised valves from all 8 patients with positive Q fever PCR results. That was primarily because our study population included patients who underwent heart valve surgery due to vegetation-associated cardiac complications. Thus, these patients are not representative of the population to which the test will be applied in clinical practice. In addition, heart valves tended to have larger vegetations before anti-*Coxiella* therapy.^[16]^ Since none of the 8 patients with a positive Q fever PCR result for excised valve tissues received anti-*Coxiella* therapy, and all underwent transesophageal echocardiography before valvular surgery, these factors might (at least partially) contribute to detection of valvular lesions.

IE is usually identified histologically by observation of vegetation and inflammatory reactions in valve tissues.^[12]^ However, these major histologic features of endocarditis are minimal or absent from patients with Q fever endocarditis.^[12]^ In general, the diagnostic pathologic feature of Q fever in liver or bone marrow samples is a fibrin ring/doughnut granuloma, defined as a small, non-necrotizing granuloma with a ring-like structure composed of fibrinoid material, often with a central fat vacuole.^[18]^ Here, we observed clusters of multinucleated giant cells, without a fibrin ring, in half of patients with a positive Q fever PCR result; no multinucleated giant cells were observed in the control Q fever-negative group. This suggests that patients with Q fever endocarditis might not develop an effective cellular immune response against the bacterium, in contrast to that observed in the liver or bone marrow during acute Q fever. Pathologic findings of histiocytic aggregation of multinucleated giant cells in valvular vegetations should lead to suspicion of Q fever. Further studies are needed to compare the histologic characteristics of various tissues from patients with Q fever along with the role of immunohistochemical analysis as a specific tool that can aid detection of *C burnetii* in tissues.^[16]^

This study has some limitations. First, we did not examine *C burnetii* DNA by conducting PCR of fresh heart valve specimens. In cases of chronic Q fever, diagnostic value increases significantly when the analysis is performed using fresh specimens.^[19]^ Therefore, our positive PCR data regarding the presence of *C burnetii* DNA in formalin-fixed heart valve tissues warrant further prospective study to examine the utility of PCR for detecting *C burnetii* DNA in fresh heart valve specimens taken from patients with suspected Q fever endocarditis. Second, we did not perform serological tests for all patients (only 2 patients were tested, and both had a low antibody titer of < 1:16). Some may argue that these 2 patients with a low antibody titer have a low probability of Q fever IE; however, a diagnosis of Q fever cardiovascular infection should not be excluded in patients with low titers of phase I IgG when they present with valvulopathy^[20]^; the use of Q fever PCR to test valve tissues would be an effective alternative to serological tests in cases in which cardiac valves are removed. Third, some may question the lack of serological *Coxiella* test in most enrolled patients with culture-negative IE during the study period given that Q fever IE has been reported as the most common cause of culture-negative IE in various regions.^[6,7]^ An annual number of reported patients with Q fever in South Korea was <20 cases every year until 2014, although the recent reported cases has increased up to 27 cases in 2015, and 26 cases by June 2016 (Supplemental Fig. 2). Furthermore, to our best knowledge, there have been only 2 reported cases of Q fever endocarditis in South Korea.^[21,22]^ Therefore, many Korean physicians did not suspect Q fever IE in the clinical setting of culture-negative IE during the study period because of low incidence of Q fever in Korea. However, we hypothesized that the under-reporting or unawareness of Q fever IE by Korean physicians might partially contribute to the low prevalence of Q fever IE in Korea. So, some cases of Q fever IE might be missed in our routine clinical setting during the study period. This is the reason why we performed this study by using Q fever PCR based on the formalin-fixed heart valve tissue to find out the missed Q fever IE cases.

In conclusion, Q fever PCR of heart valve tissues from patients who underwent heart valve surgery for blood culture–negative IE improves the diagnosis of Q fever IE. Approximately 40% of patients with culture-negative IE who underwent heart valve surgery were Q fever PCR-positive, and patients without clinical suspicion showed high mortality. Therefore, Q fever PCR analysis of heart valve tissues may increase the diagnostic yield and reduce the number of missed Q fever IE cases. This in turn will improve the clinical outcome by facilitating rapid and appropriate antibiotic therapy.

## Author contributions

**Conceptualization:** Young-Rock Jang, Joon Seon Song, Se Yoon Park, Yong Shin, Sung-Han Kim.

**Data curation:** Young-Rock Jang, Joon Seon Song, Choong Eun Jin, Byung-Han Ryu, Se Yoon Park, Sang-Oh Lee, Sang-Ho Choi, Yang Soo Kim, Jun Hee Woo, Jae-Kwan Song, Yong Shin, Sung-Han Kim.

**Formal analysis:** Young-Rock Jang, Joon Seon Song, Yong Shin, Sung-Han Kim.

**Funding acquisition:** Yong Shin, Sung-Han Kim.

**Investigation:** Young-Rock Jang, Joon Seon Song, Yong Shin, Sung-Han Kim.

**Methodology:** Young-Rock Jang, Joon Seon Song, Yong Shin, Sung-Han Kim.

**Project administration:** Se Yoon Park, Yong Shin, Sung-Han Kim.

**Resources:** Se Yoon Park, Yong Shin, Sung-Han Kim.

**Software:** Se Yoon Park, Yong Shin, Sung-Han Kim.

**Supervision:** Yong Shin, Sung-Han Kim.

**Validation:** Young-Rock Jang, Joon Seon Song, Yong Shin, Sung-Han Kim.

**Visualization:** Young-Rock Jang, Joon Seon Song, Yong Shin, Sung-Han Kim.

**Writing – original draft:** Young-Rock Jang, Joon Seon Song, Yong Shin, Sung-Han Kim.

**Writing – review and editing:** Young-Rock Jang, Joon Seon Song, Yong Shin, Sung-Han Kim.

## Supplementary Material

Supplemental Digital Content
