# Supplementary material for: Molecular detection of Coxiella burnetii in heart valve tissue from patients with culture-negative infective endocarditis
Source: Medicine (Baltimore). 2018 Aug 24;97(34):e11881. doi: 10.1097/MD.0000000000011881 (PMC6112960; doi:10.1097/MD.0000000000011881)

**Supplemental Figure 1.** **Representative histologic features of a Q fever PCR-positive patient (A, case 3) and a negative patient (B, case 4)**. (A) Clusters of multinucleated giant cells, without a fibrin ring, are visible (H & E, frozen section; original magnification, ×400). (B) Necrotizing inflammation is noted in the Q fever PCR-negative patient (H & E; original magnification, ×400).


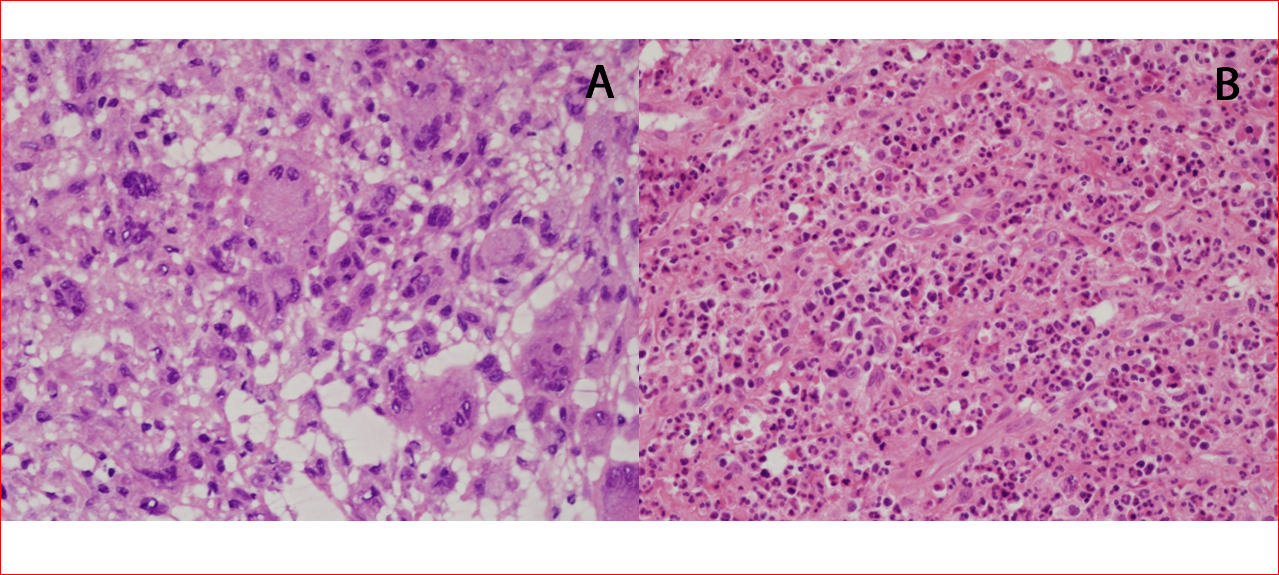


**Supplemental Figure 2.** **Reported cases of human Q fever in South Korea, from January 2001 to June 2016.**


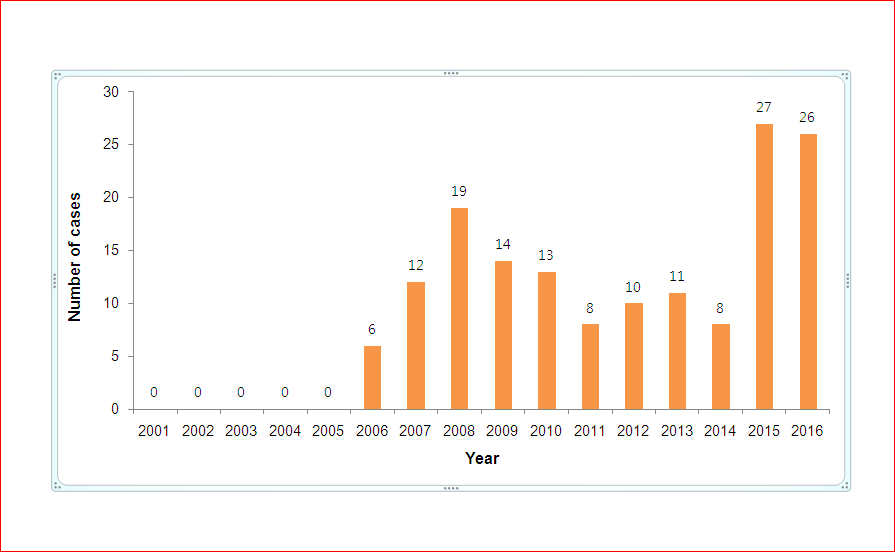

Supplement: Supplemental Digital Content [file medi-97-e11881-s001.doc]
